# The Relationship Between K-Workers’ Leader–Member Exchange, Organizational Citizenship Behavior and Task Performance—Evidence From Chinese Hospitals

**DOI:** 10.3389/fpsyg.2021.625584

**Published:** 2021-07-08

**Authors:** Xiaoli Che, Zhecheng Guo, Qinyuan Chen

**Affiliations:** ^1^School of International Studies (SoIS), Ghazali Shafie Graduate School of Government (GSGSG), Universiti Utara Malaysia, Sintok, Malaysia; ^2^Ghent Experimental Psychiatry (GHEP) Lab, Department of Psychiatry and Medical Psychology, Ghent University, Ghent, Belgium

**Keywords:** Chinese hospital, k-worker, leader–member exchange, organizational citizenship behavior, task performance

## Abstract

Aiming to reduce the difficulty of managing and motivating knowledge workers (k-workers), and promote the psychological well-being of them in Chinese hospitals, this study examines how k-workers’ leader–member exchange (LMX) influences their task performance and the mediation effect of organizational citizenship behavior (OCB). Through a self-administered survey, valid questionnaires were collected from 384 k-workers in Chinese hospitals, and partial least squares structural equation modeling was employed for data analysis. The findings show that LMX is positively related to OCB and task performance, and that OCB mediates the relationship between LMX and task performance. This research has theoretical implications and also provides practical suggestions on how to manage, motivate, and inspire k-workers, and promote the psychological well-being of them, and finally enhance the organizational performance in Chinese hospitals.

## Introduction

For the past 20 years, the most valuable asset of most organizations has been the knowledge worker (k-worker). K-workers apply their knowledge to promote the development of an organization (Henard and McFadyen, 2008). In the twenty-first century, k-workers are key to building a competitive edge, and they are responsible for finding new solutions for business development (Igielski, 2017). Hence, how to improve k-workers’ performance to enhance the core competitiveness of Chinese hospitals and better serve the public has become an urgent problem (Mei et al., 2014). To enhance the organizational performance and the development of Chinese hospital, the task performance of k-workers needs to improve. The literature indicates that increasing the degree of k-workers’ leader–member exchange (LMX) is an effective and useful way to improve their working performance (Byun et al., 2017; Lee et al., 2019). However, scholars have given very limited attention to the relationship between k-workers’ LMX and task performance in Chinese hospitals, and little is known about the mechanism underlying this relationship. Besides, the previous researches of motivating and managing the k-workers working in hospitals are usually conventional methods, such as salary incentives, promotion incentives or improving the working environment (Razzaq et al., 2019). However, very limited researches have applied the supervisor-subordinate perspective to rethink the incentive methods based on the Chinese hospital context, and put forward more useful methods to manage, maintain and motivate the k-workers working in Chinese hospitals.

In recent years, organizational managers have realized the importance of k-workers and adopted specific approaches to recruit, maintain, and motivate this kind of special and valuable resource, such as improving their pay level and giving them more attention (Morgan et al., 2016). Although the existing measures are somewhat helpful, further measures still need to be studied (Bieńkowska and Ignacek-Kunicka, 2020). In addition, Mládková et al. (2015) suggested that misdirected motivation can negatively influence k-workers’ performance, even though they are usually responsible professionals. The authors specifically identified several motivational or behavioral factors that can influence k-workers’ task performance, many of which concern the relationship between k-workers and their supervisor: examples include “does my supervisor, or someone at work, seem to care about me as a person” and “conflict between what my manager says and what he does.” Hence, the relationship between supervisor and k-workers is a crucial element that can motivate or influence k-workers’ performance (Bieńkowska and Ignacek-Kunicka, 2020). Since the relationship between supervisor and subordinate can be tested by LMX, this study will investigate the relationship between k-workers’ LMX and task performance in Chinese hospitals. LMX is a psychological variable originated from Social Exchange Theory, which can reflect the inner relationship between supervisors and subordinates. Hence, LMX is an important index in establishing the psychological well-being of k-workers in Chinese hospitals. In addition, limited researches putted the focus on how LMX can change k-workers’ behavior on Chinese hospital, so it remained unclear explanation on the path of how LMX can affect k-workers’ task performance Hence, this study will also consider the potential mediation of this relationship by organizational citizenship behavior (OCB).

## Literature Review

### K-Workers in Hospital

The “k-worker” concept was proposed by Drucker (1994). He defined k-workers as those people who are mastering and applying symbols and concepts and working with knowledge and information.

K-workers usually have a high educational level and professional knowledge and skills. These skills are characterized by high demand, short life cycles, and criticality to the organization, and include symbolic analysis skills (Despres and Hiltrop, 1995; Lee and Maurer, 1997; Jie et al., 2006), information analysis ability, distribution ability, production capacity (Banerjee, 2006), and the ability to use tools or techniques (Barley and Orr, 1997; Kubo and Saka, 2002). Reich (1991) defined k-workers as “symbolic analysts” including problem solvers who manipulate output to meet customer needs or who help to identify those needs in the market, as well as brokers such as financiers or researchers. In China, Jie et al. (2006) defined k-workers as individuals with high educational level, professional skills, and the ability to apply these skills to identify and solve problems.

Based on the hospital context, most of the clinical staff belong to k-workers (i.e., doctors, nurses). These k-workers are not engaging in simple or mechanical manual work, but complex or creative work. They are facing with complex and changing working environment because different patients have different pathological conditions (Wei, 2013). It requires k-workers who working in hospital should obtained qualified and professional medical knowledge to deal with many terrible situations in any time. Hence, the characteristics of k-workers determine that hospital managers must choose management strategies different from normal employees (Li and Song, 2012). Besides, as mentioned by Bieńkowska and Ignacek-Kunicka (2020), k-workers are usually have a strong personality, and need more autonomy when they are working. It also brings more challenges to hospital to manage when so many k-workers need to work together with different personality, cultural background and educational background (Razzaq et al., 2019). Therefore, it is great meaningful to put the focus on the k-workers in the hospitals, and manage or motivate them with more creative and useful management methods.

### Leader–Member Exchange

Derived from social exchange theory, LMX refers to the quality of exchange relationships between leaders and subordinates and illustrates how leaders form different exchange relationships with different followers in the same group over time (Dansereau et al., 1975). The LMX model provides an alternative way to understand the superior–follower relationship. This model is based on the concept that the development of a role naturally leads to differences in role definitions and various exchanges between leader and members.

Most LMX studies assume a degree of negotiating latitude between leaders and members. Subordinate positions and LMX efficiency are split into two categories: in-group and out-group (Wang, 2016). The in-group is characterized by high-quality LMX with high trust and formal or informal incentives. In-group members within the organization can receive more feedback and their work has a greater degree of freedom to contribute outside their formal responsibilities (Liden and Graen, 1980). By contrast, the out-group is characterized by low-quality LMX with low trust, support, and incentives. The interaction of out-group members within the organization are confined to the requirements of their contract of employment, meaning that they only engage in routine daily activities and interact formally with their supervisor (Lee et al., 2019).

Based on the hospital context, LMX is crucial element in the management of medical staff. According to the Donohue-Porter et al. (2019), understanding the emphasis on relationships inherent in the LMX communications helps nurse managers influence work satisfaction and organizational commitment. LMX is the key component to manage the nurses because nurses are the cornerstones of nursing administration and must be able to communicate effectively. Higher quality relationships with immediate supervisors are associated with greater structural and psychological empowerment for nurses, leading to greater psychological well-being and job satisfaction (Donohue-Porter et al., 2019). Hence, the LMX literature from hospital context shows that since LMX is an important variable in medical staff management, and LMX is associated with many positive variables, it will be possible that LMX has positive relationship on good behaviors (i.e., OCB) and well performance (i.e., task performance).

### Organizational Citizenship Behavior

The concept of OCB was introduced by Bateman and Organ (1983). Organ (1988) defined OCB as an individual behavior that is discretionary, not directly or explicitly recognized by the formal reward system, and that in aggregate promotes the effective functioning of the organization. This is the most cited definition of OCB in the literature. OCB includes activities or acts that are perceived to be extra-role, rather than in-role, and must be discretionary in nature, meaning that they are not mandatory requirements of the job (Organ, 1997; Indarti et al., 2017). Thus, performing such actions would not formally rewarded, but failure to perform such an act would not necessarily warrant punishment. Some examples of OCB include helping a colleague with their duties, engaging enthusiastically in company events, and tolerating temporary inconvenience without grievances.

Smith et al. (1983) suggested dividing OCB into two dimensions: OCB-individual (OCB-I) and OCB-organization (OCB-O). OCB-I is characterized by the quality of “altruism” intended to help a specific person, such as the leader, a workmate, or a customer. OCB-O is characterized by “general compliance,” which is seen as more impersonal and intended to benefit the organization as a whole, such as fair use of work time.

Based on the hospital context, OCB are found to have association with many positive outcome variables. The result investigated by Subhadrabandhu (2012) showed that OCB positively related to higher job satisfaction and organizational commitment among 296 hospital staff. Besides, since hospitals are non-profit organizations, how to improve the service quality of medical staff has been a difficult problem in recent years. Sidin and Arifah (2019) evidenced that the higher degree of OCB can improved the service quality of medical staff in public hospitals. Hence, the OCB literature from hospital context shows that OCB can reflect the working attitude and the working quality of medical staff, and it provides a possible explanation of how psychological change can affect medical staff’s behavior to this study. Since OCB positively relate to some active variables in some hospital context, it also implies that OCB possibly connect to the individual performance (i.e., task performance) of medical staff.

### Task Performance

Task performance refers to workers’ performance of duties officially accepted as part of their jobs. These actions contribute to the organization’s overall performance both directly (e.g., executing part of a technological process) and indirectly (e.g., supplying required materials or services) (Borman and Motowidlo, 1993).

Researchers have investigated many situational factors that can facilitate or impede task performance. The focuses of previous studies include human resource practice is positive on job crafting and task performance (Guan and Frenkel, 2018), the influence of respectful engagement and work engagement on task performance (Basit, 2019), the effects of a positive working environment and work engagement on task performance (Geue, 2018), and the effects of employee involvement climate on task performance (Smith et al., 2018).

Based on the hospital context, in recent years, the researches of the task performance by medical staff have been become a focus. Carlisle et al. (2019) investigated that many external factors can affect the task performance of medical staff, like training effectiveness and work environment. Besides, the relationships between leaders and nurses also have significant impact on their task performance. Habib et al. (2020) point out the importance of keeping an effective communication and a harmonious relationship between leader and nurse in the hospital. The positive relationships between interpersonal leadership, engagement and task performance are highlighted. Hence, the task performance literature from hospital context shows that many active variables benefit to the development of the task performance of medical staff, and it provides an information to this study that a good interpersonal relationship may acts as a key role on the improving of the task performance of medical staff. Therefore, it implies that the relationship between supervisors and subordinates possibly connect to the task performance of medical staff.

## Theoretical Framework

**FIGURE 1:**
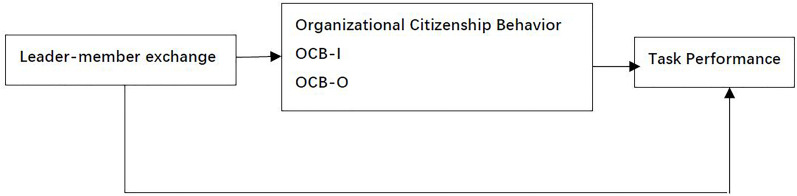
Theoretical framework.

A theoretical framework is used to model how and explain why particular phenomena, variables, or concepts are related to one another (Sekaran and Bougie, 2010). Drawing on social exchange theory, this study tests the relationship between k-workers’ LMX and task performance and the mediating roles of OCB-I and OCB-O. The following Figure 1 shows the theoretical framework of this research.

### Leader–Member Exchange and Organizational Citizenship Behavior

In the context of social exchange and reciprocity, the primary reason for the relationship between LMX and OCB was investigated (Blau, 1964). Blau (1964) suggested that social interactions are built on trust and the hope that one individual will reciprocate another’s acts of goodwill. When certain actions by the supervisor are perceived positively by subordinates (high-quality LMX), these actions evoke feelings of subordinate obligation (Harris et al., 2014; Chow et al., 2015; Tang and Naumann, 2015). Subordinates thus respond by engaging in extra-role behavior (e.g., OCB) as a way of fulfilling the perceived duty.

The related studies based on hospital context have been investigated. Zhang et al. (2020) used structural equation modeling to prove that LMX has positive relationship with OCB among 426 nurses recruited from 12 public hospitals in China. They point out that LMX and OCB are crucial in evaluating the attitude of medical employees. Besides, according to Yusof et al. (2019), the positive relationships between LMX and OCB existed in the 539 medical employees who working in 133 public hospitals. This research highlighted that the personal-based relationship between the leader and the subordinate needs to be practiced in order to improve the chances of implementing OCB. Hence, according to previous researches, this study proposes the following hypotheses:

**H1**: There is a positive relationship between k-worker’s LMX and OCB in Chinese hospitals.
H1a: There is a positive relationship between k-worker’s LMX and OCB-I in Chinese hospitals.
H1b: There is a positive relationship between k-worker’s LMX and OCB-O in Chinese hospitals.

### Organizational Citizenship Behavior and Task Performance

According to Borman and Motowidlo (1993), OCB can improve performance because it is the “lubricant” of the social machine in an organization. Hanafi et al. (2018) suggested that the existence of such behavior will improve employees’ motivation at work, facilitate social communication among employees, reduce disputes, improve work efficiency, and ultimately improve employees’ performance.

The related studies based on hospital context has been investigated. Wibowo and Mochklas (2020) explained the urgency of OCB toward performance of nurses of type C hospitals in Surabaya because OCB not only can enhance productivity of work partners, but also can help resources saving to maintain group functions. As a huge individual contribution, OCB exceed the demands of roles in the organization, which made the achievement of excellent performance in the hospitals. Besides, Udin and Yuniawan (2020) tested the relationships between psychological capital, personality traits of big-five, OCB and task performance among 246 employees. It highlighted that OCB application in the working environment could develop the performance of the individual employee, performance of the unit, and performance of the organization. Hence, according to previous researches, this study proposes the following hypotheses:

**H2**: There is a positive relationship between k-worker’s OCB and task performance in Chinese hospitals.
H2a: There is a positive relationship between k-worker’s OCB-I and task performance in Chinese hospitals.
H2b: There is a positive relationship between k-worker’s OCB-O and task performance in Chines hospitals.

### Leader–Member Exchange and Task Performance

There are several reasons to expect a positive relationship between the quality of LMX and followers’ task performance. First, the leader provides tangible and intangible benefits to followers in a high-quality leader–member relationship (Kuvaas and Buch, 2018). Tangible benefits (e.g., resources, money) promote better performance by offering them more profit and wealth, while intangible benefits (e.g., favors, appreciation, affection from the superior) motivate subordinates to reciprocate by making extra efforts or dedicating themselves to their work (Kuvaas et al., 2012). Second, followers in a high-quality relationship with their leader receive more support, are more motivated, and have a higher degree of job satisfaction compared to subordinates with low-quality LMX (Gerstner and Day, 1997; Hill et al., 2014). Third, followers in high-quality LMX relationships have greater opportunities for advancement compared to subordinates in low-quality LMX relationships. Therefore, they are more inclined and driven to perform well.

The related studies based on hospital context have been investigated. Nasiatin et al. (2021) used Partial Least Square to confirm the positive relationship between knowledge sharing, LMX, OCB and employee performance in the hospital. The research pointed out that LMX has a positive effect on individual performance, and finally contribute to the organizational performance (hospital performance). Besides, Sepdiningtyas and Santoso (2017) tested the relationship between LMX, work engagement, co-workers support and task performance among hospital nurses. The research confirmed the positive relationship between LMX and task performance and highlighted that the high quality LMX generates leader support both professionally and emotionally. This will encourage followers to offer their optimal abilities to complete their tasks, which is beneficial to the development of task performance of hospital nurses. Hence, according to previous researches, this study proposes the following hypotheses:

**H3**: There is a positive relationship between k-worker’s LMX and task performance in Chines hospitals.

### Leader–Member Exchange, Organizational Citizenship Behavior, and Task Performance

OCB is anticipated to mediate the LMX–task performance relationship because high-quality LMX entails an emotional connection and generally unstated shared expectations of reciprocity. In a high-quality exchange relationship, supervisors cater to subordinates’ higher social needs by guiding them to prioritize collective benefits over individualized satisfaction in the short term. “Good citizens” (high OCB) are more willing to pursue collective interests. Hence, employees with high-quality LMX are more committed to performing citizenship behaviors to promote the development of their organization in return (Truckenbrodt, 2000; Chow et al., 2015).

OCB is typically voluntary and not paid. Individuals performing OCB tend to show altruism, organizational commitment, and conscientiousness (LePine et al., 2002; Rapp et al., 2013), which are all positively related to task performance (Rapp et al., 2013). Accordingly, it is reasonable to expect a positive correlation between OCB and task performance. Hence, based on previous researches, this study proposes the following hypotheses:

**H4**: OCB mediates the relationship between k-worker’s LMX and task performance in Chines hospitals.
H4a: OCB-I mediates the relationship between k-worker’s LMX and task performance in Chines hospitals.
H4b: OCB-O mediates the relationship between k-worker’s LMX and task performance in Chines hospitals.

## Research Method

### Sampling

According to Sekaran and Bougie (2003), the sample should include an adequate number of suitable individuals from the target population in order to estimate the parameter of the population. This research specifically focuses on k-workers working in 10 Chinese hospitals (including doctors, nurses, administrative staff, etc.). According to Jie et al. (2006), k-workers are a special group of people with at least a bachelor degree. Hence, this study only recruited people with a bachelor degree or higher educational qualification.

### Data Collection Method

Data were collected using a self-administered questionnaire that was distributed to k-workers in Chinese hospitals. Measurement items in the questionnaire were adapted from previous researches. Of the 600 questionnaires distributed, 384 valid questionnaires were returned. Krejcie and Morgan (1970) suggested that for a population of over 100,000 people, a sample size of 380 is sufficient. Therefore, 384 k-workers from 10 Chinese hospitals were considered sufficient for this study.

The LMX measure was adapted from Graen and Uhl-Bien (1995) and applied to the hospital context. Participants responded to the seven items. on a 5-point Likert scale ranging from 1 (“strongly disagree”) to 5 (“strongly agree”). The sample items are: “I usually know how satisfied my supervisor is with what I do in the hospital. My supervisor recognizes my potential in the hospital. I would defend and justify my supervisor’s decision if he/she were not present to do so in the hospital.”

The OCB scale was adapted from Smith et al. (1983) and applied to the hospital context. It comprised seven items for OCB-I and nine items for OCB-O. Participants responded to each item on a 5-point Likert scale ranging from 1 (“strongly disagree”) to 5 (“strongly agree”). The sample items are: “I help other employees with their work when they have been absent in the hospital. I help others when their work load increases (assist others until they get over the hurdles) in the hospital. I volunteer to do things not formally required by the job in the hospital.”

The task performance (TP) measure was adapted from Koopmans et al. (2014) and applied to the hospital context. Participants responded to seven items on a 5-point Likert scale ranging from 1 (“strongly disagree”) to 5 (“strongly agree”). The sample items are: “I managed to plan my work so that it was done on time in the hospital. I kept in mind the results that I had to achieve in my work in the hospital. I was able to perform my work well with minimal time and effort in the hospital.”

### Data Analysis Techniques

The data collected from the self-administered questionnaires were analyzed using partial least squares structural equation modeling (PLS-SEM). PLS facilitates the analysis of a set of interrelated research questions by modeling the relationships among multiple constructs (Anderson and Gerbing, 1988). Even though covariance-based approach (CB-SEM) such as AMOS has been a focused by previous researches (Hair et al., 2013), however, a variance-based approach or PLS-SEM with distinctive methodological attributes making it a more possible alternative to the popular CB-SEM approach (Henseler et al., 2009). Besides, when employing PLS-SEM, it is a benefit that the study can get high efficiency in parameter estimation. This is because it demonstrated in the technique’s superior statistical power compare to CB-SEM. Greater statistical power refers to the PLS-SEM is more likely to provide a specific relationship significant when it is, in fact, significant in the population (Hair et al., 2017). Thus, PLS-SEM is more appropriate to test the study’s hypotheses than CB SEM as it is a flexible and good technique to build a statistical model and make a prediction (Ringle et al., 2010). Hence, the data will be analyzed by using Statistical Package for Social Sciences (SPSS) version 20.0 and SmartPLS 2.0 M3 software.

## Results

### Demographic PROFILE

Table 1 presents the demographic characteristics of the study’s respondents.

**TABLE 1:** Demographic characteristics of the respondents.

**Demographics**	**Category**	***n***	**%**
Gender	Male	182	47.4
	Female	202	52.6
Age	<30	35	9.1
	30–39	123	32.0
	40–50	140	36.5
	>50	86	22.4
Education	Bachelor degree	231	60.2
	Master degree or above	153	39.8
Position	Base level	198	51.6
	Middle level	177	46.1
	High level	9	2.3
Occupation	Doctor	135	35.2
	Nurse	107	27.9
	Administrative staff	112	29.1
	Contingent worker	30	7.8

Most respondents are female (52.6%) and all respondents were aged between 28 and 55 years old, with the largest group (36.5%) aged between 40 and 50. In order to screen out the target respondents, education background is the main criterion. The participants who have a bachelor degree account for 60.2%, while the remainder have at least a master degree. Besides, regarding position in the organization, 51.6% of employees were at base level, while 46.1% were at middle level. Finally, regarding to the occupation in the hospital, based on the type of work, there are total 35.2% of participants is doctor, 27.9% is nurse, 29.1% is administrative stuff and the rest is contingent worker (7.8%).

### Composite Reliability and Convergent Validity

Evaluating the measurement model is the first step in PLS-SEM. The validity of the measurement model needs to be determined before checking a model or performing hypothesis testing. This includes checking that the instruments actually measure what they are intended to. First, composite reliability needs to be tested because reliability and validity are related. Although high reliability does not assure high validity, low reliability means that validity cannot be high. According to Hair et al. (2017), composite reliability values over 0.7 are considered reliable. As shown in Table 2, the composite reliability values of the four variables, LMX, OCB-I, OCB-O, and TP, are all over 0.7, thus meeting the requirement for establishing composite reliability.

**TABLE 2:** Composite reliability results.

**Model construct**	**Items**	**Composite reliability**
LMX	7	0.967
OCB-I	7	0.945
OCB-O	9	0.928
TP	7	0.958

*Where the “LMX” abbreviation refers to leader-member exchange, OCB-I, organizational citizenship behavior—individual; OCB-O, organizational citizenship behavior—organization. The “TP” abbreviation refers to task performance.*

Second, the model’s convergent validity needs to be tested. Convergent validity refers to the degree of similarity of measurement results when different measurement methods are used to determine the same feature. According to Hair et al. (2017), to establish acceptable convergent validity, the factor loading needs to exceed 0.5 and the average variance extracted (AVE) needs to exceed 0.5. As Table 3 shows, the factor loadings of LMX, OCB-I, OCB-O, and TP all exceed 0.5, and the four variables’ AVE values all exceed 0.5. These results confirm that all four variables have acceptable convergent validity.

**TABLE 3:** Convergent validity results.

**Model construct**	**Measurement items**	**Loading**	**AVE**
LMX	LMX1	0.928	
	LMX2	0.905	
	LMX3	0.929	0.808
	LMX4	0.815	
	LMX5	0.927	
	LMX6	0.880	
	LMX7	0.901	
	OCB-I1	0.856	
	OCB-I2	0.798	
	OCB-I3	0.879	
	OCB-I4	0.870	
OCB-I	OCB-I5	0.843	0.710
	OCB-I6	0.786	
	OCB-I7	0.861	
	OCB-O1	0.806	
	OCB-O2	0.808	
	OCB-O3	0.755	
	OCB-O4	0.799	
	OCB-O5	0.735	
OCB-O	OCB-O6	0.731	0.590
	OCB-O7	0.785	
	OCB-O8	0.714	
	OCB-O9	0.771	
TP	TP1	0.824	
	TP2	0.867	
	TP3	0.928	0.767
	TP4	0.897	
	TP5	0.862	
	TP6	0.886	
	TP7	0.863	

*Where the “LMX” abbreviation refers to leader-member exchange, OCB-I, organizational citizenship behavior—individual; OCB-O, organizational citizenship behavior—organization. The “TP” abbreviation refers to task performance.*

### Discriminant Validity

Discriminant validity requires that the observed values should be distinguishable from one another when different constructs are measured using different methods. According to Fornell and Larcker (1981), discriminant validity is acceptable when the square root of the AVE of a construct is higher than the correlation between that construct and other constructs. As Table 4 shows, all four constructs meet this criterion. These results confirm that all four variables have acceptable discriminant validity.

**TABLE 4:** Discriminant validity of constructs.

**Construct**	**LMX**	**OCB-I**	**OCB-O**	**TP**
LMX	**0.899**			
OCB-I	0.695	**0.843**		
OCB-O	0.713	0.543	**0.768**	
TP	0.491	0.665	0.666	**0.876**

*Where the “LMX” abbreviation refers to leader-member exchange, OCB-I, organizational citizenship behavior—individual; OCB-O, organizational citizenship behavior—organization. The “TP” abbreviation refers to task performance. The bold value means the square root of AVE of each variable.*

### Analysis of the Structural Model

**FIGURE 2:**
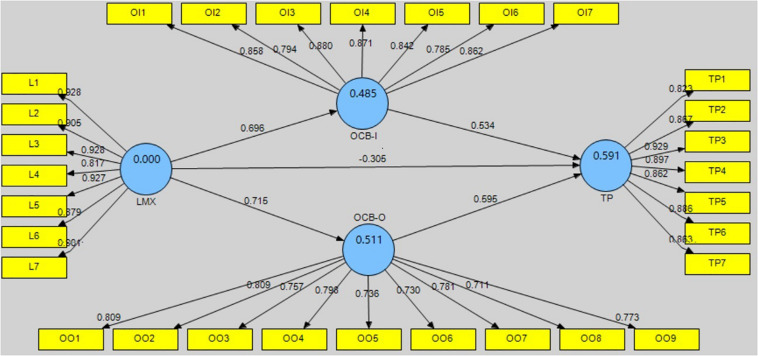
The first order structural model for individual latent variable.

**FIGURE 3:**
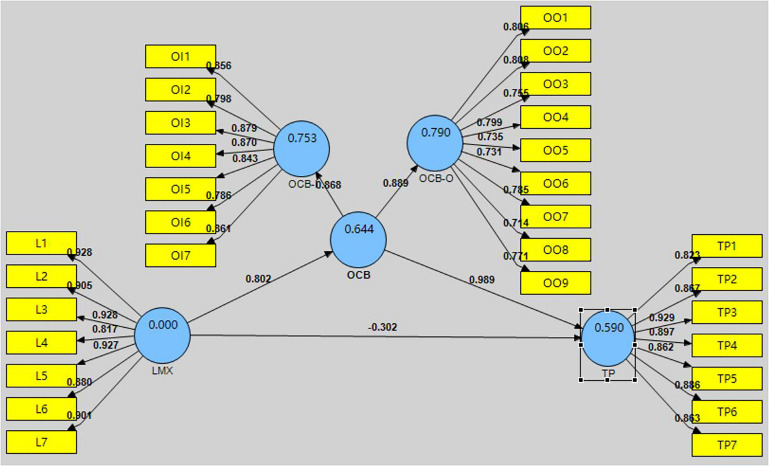
The second order structural model for main latent variable.

*R*^2^-values can be used to investigate the quality of each variable in the structural model. If *R*^2^ is within the range 0–1 then it is acceptable (Hair et al., 2017). Figure 2 shows the first order of structural model while the Figure 3 shows the second order of structural model. As Figure 2 shows, the endogenous variables of OCB-I and OCB-O are at a substantial level, with *R*^2^-values of 0.485 and 0.511, respectively. The *R*^2^*-*value of TP is 0.591, which is also acceptable. As Figure 3 shows, OCB, OCB-I, and OCB-O are at a substantial level, with *R*^2^-values of 0.644, 0.753, and 0.790, respectively. The *R*^2^-value of TP is 0.590, which is also acceptable.

### Goodness of Fit

In PLS the global standard of goodness of fit (GoF) is applied to measure the entire model. GoF is calculated as the geometric mean of the average communality and average *R*^2^ of the endogenous constructs. Following Wetzels et al. (2009), the model was evaluated with the baseline values of GoF (small = 0.1, medium = 0.25, large = 0.36). As Table 5 shows, the model’s GoF was 0.533, which indicates adequate PLS model validity.

**TABLE 5:** Structural model specification.

**Construct**	***R*^2^**	**Communality**
LMX	Predictor	0.806
OCB	0.645***	0.496
TP	0.591***	0.767
Σx/n	0.412	0.690
[(Σ*x*R2)/n] × [(Σ*xComm/n*]		0.284
Goodness of fit (GoF)		0.533***

According to Wetzels et al. (2009), GoF_*small*_ = 0.10*, GoF_*medium*_ = 0.25**, and GoF_*large*_ = 0.36***. Where the “LMX” abbreviation refers to leader-member exchange, OCB-I, organizational citizenship behavior—individual; OCB-O, organizational citizenship behavior—organization. The “TP” abbreviation refers to task performance. The bold value means the GOF value of the main model.

### Hypothesis Testing

**FIGURE 4:**
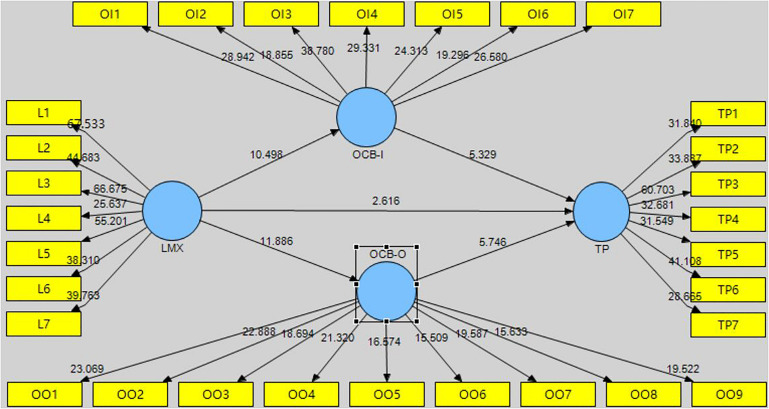
Hypothesis testing of first order model.

**FIGURE 5:**
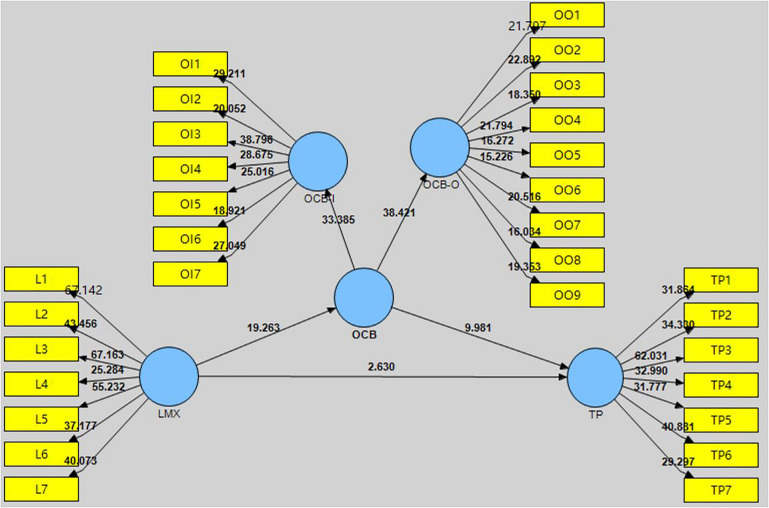
Hypothesis testing of second order model.

The hypotheses were tested using bootstrapping, which entails repeated random sampling and replacement of the original sample to create a bootstrap sample. The resulting standard error is used to verify each hypothesis. Figures 4, 5 show the PLS bootstrapping method for the first-order and the second-order of structural model.

Table 6 presents the results for the hypothesized structural relationships between LMX, OCB (OCB-I, OCB-O), and TP. A 5% significance level of the two-tailed test requires a *t*-value of at least 1.96, while a 1% significance level of the two-tailed test requires a *t*-value of at least 2.58. LMX was found to be significantly associated with TP (*t* = 5.905), OCB (*t* = 19.484), OCB-I (*t* = 10.441), and OCB-O (*t* = 11.938). OCB was found to be significantly associated with TP (*t* = 10.068). Furthermore, OCB-I and OCB-O were also significantly associated with TP (*t* = 5.238, *t* = 5.639, respectively). Thus, hypotheses H1, H1a, H1a, H2, H2a, H2b, and H3 were supported.

**TABLE 6:** Hypotheses testing.

**Hypothesis**	**Relationship**	**Standard error**	**T statistics**	**Supported**
H1	LMX—OCB	0.041	19.484**	Yes
H1a	LMX—OCB-I	0.067	10.441**	Yes
H1a	LMX—OCB-O	0.060	11.938**	Yes
H2	OCB—TP	0.098	10.068**	Yes
H2a	OCB-I—TP	0.102	5.239**	Yes
H2b	OCB-O—TP	0.106	5.639**	Yes
H3	LMX—TP	0.083	5.905**	Yes

*Significant at p < 0.05 at a two-tailed T statistics value of 1.96; **Significant at p < 0.01 at a two-tailed T statistics value of 2.58. Where the “LMX” abbreviation refers to leader-member exchange, OCB-I, organizational citizenship behavior—individual; OCB-O, organizational citizenship behavior—organization. The “TP” abbreviation refers to task performance.

To test the mediating effects of OCB, OCB-I, and OCB-O on the relationship between LMX and task performance, this study assessed how much of the direct path is absorbed by calculating variation accounted for (VAF): VAF < 0.2 means no mediation effect, VAF from 0.2 to 0.8 means partial mediation, and VAF > 0.8 means full mediation. As Table 7 shows, OCB, OCB-I, and OCB-O fully mediate the relationship between LMX and TP. Hence, H4, H4a, and H4b were supported.

**TABLE 7:** Testing the mediation effect.

**Hypothesis**	**Relationship**	**VAF**	**Supported**
H4	LMX—OCB—TP	1.620	Yes
H4a	LMX—OCB-I—TP	0.833	Yes
H4b	LMX—OCB-O—TP	0.939	Yes

*Where the “LMX” abbreviation refers to leader-member exchange, OCB-I, organizational citizenship behavior-individual; OCB-O, organizational citizenship behavior-organization. The “TP” abbreviation refers to task performance.*

## Discussion

Taking k-workers working in Chinese hospitals as the research object and analyzing data collected through a questionnaire survey, this research investigated the relationships among LMX, OCB (OCB-I, OCB-O), and task performance from both theoretical and empirical perspectives. The results showed that LMX is positively related to task performance, which is consistent with previous research findings (Wang, 2016; Lee et al., 2019). In detail, the leader provides extra benefits to the follower in a high-quality supervisor-subordinate dyad. Hence, based on the principle of reciprocity on the social exchange theory, these benefits allow k-workers to make extra efforts or dedicate themselves to their work, which is benefit to the achievement of better task performance. This finding indicates that high-quality LMX and a harmonious relationship between supervisor and subordinates can promote increased task performance of k-workers in Chinese hospitals, ultimately contributing positively to long-term organizational success. Therefore, the present study suggests that LMX could be used as a basis for recruiting and selecting public sector supervisors. For example, nice and kind supervisors can create a harmonious relationship with their k-worker followers.

The results also showed that LMX was positively related to OCB and to its two dimensions of OCB-I and OCB-O, consistent with the findings of Harris et al. (2014), Chow et al. (2015), and Tang and Naumann (2015). This finding indicates that high-quality LMX leads k-workers to perceive that their superior trusts and supports them. To maintain this social exchange relationship, based on the principle of reciprocity on the social exchange theory (Blau, 1964), subordinates will try to work harder and even undertake tasks beyond their job requirements. Besides, the significant relationship between LMX and OCB also indicates that in a high-quality exchange relationship, the leader offers precious incentives for followers, including rewards for going beyond their formal duties. Therefore, according to social exchange theory, to preserve a balanced or equal relationship of exchange, subordinates are more likely to perform extra-role behaviors (Huang et al., 2014). Good relationships between leaders and k-workers in Chinese hospitals is vital to motivating k-workers to carry out more extra-role behaviors.

Furthermore, this study empirically evidenced that k-workers’ OCB (OCB-I, OCB-O) mediated the relationship between LMX and task performance in Chinese hospitals. According to the principle of reciprocity on the social exchange theory, when LMX between leaders and k-workers is high quality, subordinates are more likely to engage in OCB as return, which in turn helps to promote their task performance and, ultimately, benefits organizational performance. Thus, this result may indicate that managers need to establish and maintain harmonious relationships with their subordinates. It is also advisable to consider LMX and OCB in performance evaluations because both are directly related to k-workers’ task performance.

With a high-quality degree of LMX with their supervisors, k-workers tend to establish a stabilized and harmonious relationship with supervisors. It will be benefit of the development of a psychological well-being. Such a psychological well-being is the key to the drive of engaging in OCB because of the autonomy of OCB and, finally, benefits task performance. Besides, such a psychological well-being also has a direct effect on improving the task performance of k-worker. Hence, starting from the supervisor-subordinate dyads perspective, the inner social exchange is the origin of k-worker’s psychological well-being and ultimately benefits organizational development.

Overall, followed the suggestion of Hill et al. (2016) that more study should be investigated in different group, this study has some theoretical contribution and practical contribution. Theoretically, based on social exchange theory, this study established a theoretical framework that explained the relationship between k-worker’s LMX, OCB and task performance. It contributes to the LMX literature with a special group and different cultural background. Practically, this research provided some useful implication to hospital managers, which is helpful for them to manage and motivates their k-worker employees. Such implications indicated that LMX is the key of management in hospital because the degree of LMX associate with the k-worker’s psychological well-being, and finally connect to the organizational performance in hospital.

## Limitations

This study has two main limitations. First, both the independent variable (LMX) and the mediator (OCB) were measured in the same group and at the same time, so common method bias cannot be entirely ruled out. To address this limitation, future studies should collect data on the independent variable and mediator at different times. Second, although the data were collected from a convenience sample of k-workers in several Chinese hospitals, the sample size still relatively small. Therefore, the findings are limited to the studied sample and cannot be generalized to all Chinese hospitals. More research should be conducted in different kinds of working group and in different cultural contexts. Finally, since this research adapted the scale from Graen and Uhl-Bien (1995) to test the k-worker’s degree of LMX, only subordinates are needed to fill the questionnaires. Hence, it will be a limitation that the situation of supervisor-subordinate dyad is out of considering. The further research should be investigated in different supervisor-subordinate dyads, and such sample will be more contributive to the further study.

## Data Availability Statement

The raw data supporting the conclusions of this article will be made available by the authors, without undue reservation.

## Ethics Statement

The studies involving human participants were reviewed and approved by the Universiti Utara Malaysia. The patients/participants provided their written informed consent to participate in this study.

## Author Contributions

XC performed this research and finished the manuscript of this research. ZG analyzed the data of this research. QC edited the manuscript. All authors contributed to the article and approved the submitted version.

## Conflict of Interest

The authors declare that the research was conducted in the absence of any commercial or financial relationships that could be construed as a potential conflict of interest.
